# Surveillance Length and Validity of Benchmarks for Central Line-Associated Bloodstream Infection Incidence Rates in Intensive Care Units

**DOI:** 10.1371/journal.pone.0036582

**Published:** 2012-05-07

**Authors:** Patricia S. Fontela, Caroline Quach, David Buckeridge, Madukhar Pai, Robert W. Platt

**Affiliations:** 1 Department of Epidemiology, Biostatistics, and Occupational Health, McGill University, Montreal, Quebec, Canada; 2 Department of Pediatrics, The Montreal Children's Hospital – McGill University, Montreal, Quebec, Canada; University of Maryland, United States of America

## Abstract

**Introduction:**

Several national and regional central line-associated bloodstream infections (CLABSI) surveillance programs do not require continuous hospital participation. We evaluated the effect of different hospital participation requirements on the validity of annual CLABSI incidence rate benchmarks for intensive care units (ICUs).

**Methods:**

We estimated the annual pooled CLABSI incidence rates for both a real regional (<100 ICUs) and a simulated national (600 ICUs) surveillance program, which were used as a reference for the simulations. We simulated scenarios where the annual surveillance participation was randomly or non-randomly reduced. Each scenario's annual pooled CLABSI incidence rate was estimated and compared to the reference rates in terms of validity, bias, and proportion of simulation iterations that presented valid estimates (ideal if≥90%).

**Results:**

All random scenarios generated valid CLABSI incidence rates estimates (bias −0.37 to 0.07 CLABSI/1000 CVC-days), while non-random scenarios presented a wide range of valid estimates (0 to 100%) and higher bias (−2.18 to 1.27 CLABSI/1000 CVC-days). In random scenarios, the higher the number of participating ICUs, the shorter the participation required to generate ≥90% valid replicates. While participation requirements in a countrywide program ranged from 3 to 13 surveillance blocks (1 block = 28 days), requirements for a regional program ranged from 9 to 13 blocks.

**Conclusions:**

Based on the results of our model of national CLABSI reporting, the shortening of participation requirements may be suitable for nationwide ICU CLABSI surveillance programs if participation months are randomly chosen. However, our regional models showed that regional programs should opt for continuous participation to avoid biased benchmarks.

## Introduction

Central line-associated bloodstream infections (CLABSI) are associated with an important burden of illness in intensive care units (ICUs).[1] National and regional surveillance programs are essential to provide information on ICU CLABSI epidemiology and on changes in trends over time. Furthermore, surveillance results are used for the planning and evaluation of infection control measures, as well as for the generation of benchmarks. Despite their importance, national and regional surveillance programs face significant challenges when recruiting participating hospitals. Several hospitals cite limited resources for performing continuous surveillance as the reason for not participating in such programs.

Consequently, many national and regional ICU CLABSI surveillance programs have eliminated the continuous participation requirement from their protocols. For example, the National Healthcare Safety Network (NHSN) in the U.S. requires ICUs to participate a minimum of 1 month/year, while in England the cut-off is 3 months/year.[2], [3] In the Netherlands, hospitals participate at their own discretion in the national surveillance program.[4], [5].

Reducing the annual hospital participation in surveillance programs raises concerns about the validity of the obtained benchmarks, as the aforementioned cut-offs are arbitrary and variable. Furthermore, no study has yet evaluated the minimum number of months ICUs must participate in a national or a regional surveillance program to generate valid benchmarks for annual pooled CLABSI incidence rates. Thus, the purpose of this study was to determine, through simulation, the impact of different participation requirements on the ability of countrywide and regional surveillance programs to yield valid estimates of the true annual ICU pooled CLABSI incidence rates.

## Methods

This study was approved by the McGill University Institutional Review Board and the need for informed consent was waived.

### Data sources used for building the complete databases

To answer our research question at a national level, we simulated a database of a countrywide ICU CLABSI surveillance program containing 600 ICUs (480 adult units, 48 pediatric ICUs – PICUs -, and 72 neonatal ICUs – NICUs -; 60% teaching units) that continuously participated in the program during one year. We used published data from NHSN to model the ICU population structure (type of ICU and academic profile), and data from the Surveillance Provinciale des Infections Nosocomiales (SPIN) program, an ICU CLABSI surveillance program in the province of Quebec, Canada, to model the variables used for the calculation of CLABSI incidence rate (CLABSI cases – numerator - and central venous catheter-days – CVC-days, denominator) per participating ICU and for each of the 13 28-day surveillance blocks/year.[6], [7], [8], [9], [10], [11] A detailed explanation of the simulation model can be found in Appendix S1.

The simulation model used to create the national database was run 1000 times. This generated 1000 independent and complete databases, i.e. without missing data. For the purpose of this study, we took a random subset of 100 national simulated databases upon which we performed our statistical analyses.

We also used the SPIN database to determine the effect of different participation requirements at a regional level. We initially built a dataset with no missing values including 44 ICUs (34 adult ICUs, 4 PICUs, and 6 NICUs) that continuously participated in the SPIN program during 2007–2008 (complete dataset I).[6] Variables contained in this dataset were: type of ICU, academic profile, and number of CLABSI cases and CVC-days per surveillance period for each ICU (13 blocks/year). To check the reliability of our results, we built a second regional database, which included 53 ICUs (43 adult ICUs, 4 PICUs, and 6 NICUs) that continuously sent data to SPIN during 2008–2009 (complete dataset II).

### Calculation of the reference annual ICU pooled CLABSI incidence rates

We calculated the annual ICU pooled CLABSI incidence rate for adult, pediatric, and neonatal ICUs for all national (100) and regional (2) complete databases, using the following formula:[12]

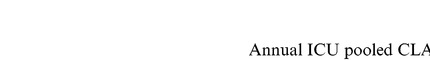
These rates were considered the “reference rates” for each database because they were calculated using 100% of the data. We then calculated intervals which limits were values 10% above and below the annual reference rates.

### Simulation scenarios for different length of participation

The complete national and regional datasets were used as the starting point for the creation of scenarios where the duration of ICU participation in the surveillance programs was progressively shortened. To do so, data were either randomly or non-randomly removed.

#### 1. Random removal of data – equal participation scenarios

In these scenarios, we simulated a situation where the surveillance program determined the ICU participation length per year, making all units participate for an equal number of blocks, but allowing ICUs to choose, in advance, when (i.e., in which blocks) data would be collected. We assumed that ICUs' choice of when to collect data was made in an independent and random way.

We started by generating a scenario where each ICU submitted data (i.e., number of CLABSI and CVC-days) for the entire year except for 1 block that was randomly chosen out of the 13 blocks. We progressively removed data from 1 additional random block per ICU until we reached 12 random blocks of missing data.

#### 2. Random removal of data – unequal participation scenarios

In these scenarios, we simulated the approach currently used by many surveillance programs. The minimal required ICU participation per year was set; however, units could choose to participate for more than the minimum and could decide during which blocks data would be collected. For the purpose of this study, the minimal participation was defined as 1 block/year. Again, we assumed that ICUs chose the surveillance blocks in an independent and random way.

First, we created a scenario where the total ICU population had an average participation of 12 surveillance blocks/year. We progressively decreased the average participation 1 block at a time, until we reached an average participation of 1 block. Blocks were randomly removed.

The generation of the equal and unequal participation scenarios was repeated 1000 times per scenario for all national and regional databases. Adult, pediatric, and neonatal ICU annual pooled CLABSI incidence rates were calculated for each iteration. We then built a distribution of the 1000 CLABSI incidence rate estimates (for adult, pediatric, and neonatal ICUs) for each complete database and calculated their expected means

The expected means calculated for the random scenarios using the regional databases were directly compared to the “reference rates”. For the random scenarios involving the national surveillance program, we first built distributions of the expected means for adult, pediatric, and neonatal ICUs calculated for each of the 100 national databases. Subsequently, we took the means of these distributions and compared them to their respective “reference rates”.

#### 3. Non-random removal of surveillance periods

These scenarios examined a situation where the surveillance program would determine not only the yearly ICUs' length of participation, but also when data should be collected. The options in which surveillance lasted 9, 6, and 3 blocks/year were evaluated. We investigated 4 different alternatives for when data were required to be collected: 1) continuous data collection for the first 9, 6, or 3 blocks, 2) the last 9, 6, or 3 blocks, 3) the 9, 6, or 3 middle blocks, and 4) alternated data collection for a total of 9, 6, or 3 blocks. Adult, pediatric, and neonatal ICU annual pooled CLABSI incidence rates were estimated for all 12 scenarios.

The annual pooled CLABSI incidence rate estimates generated for the regional surveillance program were directly compared to the “reference rates”. For the non-random scenarios involving the national surveillance program, we calculated the mean of the 100 adult, pediatric, and neonatal estimates and compared them to their respective “reference rates”.

### Simulation outcomes

Our primary outcome was defined as the validity of the estimates of adult, pediatric, and neonatal annual ICU pooled CLABSI incidence rates for a regional and a national surveillance program (see “Statistical comparisons”). An estimate was considered valid if it was within 10% of the “reference rate”. As secondary outcomes, we evaluated the estimated average bias and the proportion of valid simulated iterations.

### Statistical comparisons

All simulations were performed using R 2.11.0. The performance of our model used to simulate the national surveillance program was evaluated through the assessment of bias and root mean square error.[13], [14] To do so, we compared the mean simulated adult, pediatric, and neonatal ICU pooled CLABSI incidence rates to the SPIN adult, pediatric, and neonatal ICU pooled CLABSI incidence rates for 2007–2009.

The comparison between the estimates of the annual ICU pooled CLABSI incidence rates and the “reference rates” was performed in 3 ways:[15]

1. Validity: an estimate, including the expected mean calculated for the random data removal scenarios, was considered to be valid if it was within 10% of the “reference rate”. The 10% range was determined based on a consensus among infection control experts.
2. Average bias: calculated by subtracting the expected mean of the estimate from the “reference rate”.
3. Proportion of iterations whose annual ICU CLABSI pooled incidence rate estimates were valid: exclusive to the random data removal scenarios. A scenario was considered acceptable if the estimated rate was valid, i.e., was within 10% of the true rate, in at least 90% of the iterations.

## Results

### Performance of the model used to simulate the national surveillance program database

Estimation of the adult, pediatric, and neonatal annual ICU pooled CLABSI incidence rates presented bias of −0.23, −0.05, and −0.33 CLABSI/1000 CVC-days, respectively. The maximum amount of bias (NICU) represented a decrease of 6.6% of the true CLABSI incidence rate and was considered acceptable. Adult, pediatric, and neonatal ICU CLABSI incidence rates presented random mean square errors of 0.053, 0.119, and 0.240, respectively.

### Calculation of “reference rates”

Estimated “reference rates” rates for adult, pediatric, and neonatal ICUs using the simulated national database were 1.52, 2.13, and 4.67 CLABSI/1000 CVC-days, respectively. “Reference rates” for adult, pediatric, and neonatal ICUs at a regional level were 1.83, 2.79, and 5.69 CLABSI/1000 CVC-days for 2007–2008, respectively. For the 2008–2009 periods, “reference rates” were 1.68 CLABSI/1000 CVC-days (adult ICUs), 1.52 CLABSI/1000 CVC-days (PICUs), and 4.18 CLABSI/1000 CVC-days (NICUs).

### Random scenarios with equal ICU participation

At a national level, all scenarios presented valid estimates for all annual ICU pooled CLABSI incidence rates (bias −0.0091 to 0.0119 CLABSI/1000 CVC-days – Table 1). The minimum participation required for adult ICUs to yield 90% of valid iterations was 3 surveillance blocks, while PICUs and NICUs required participation during the entire surveillance year (13 blocks).

Similarly, all scenarios presented valid estimates for all annual ICU pooled CLABSI incidence rates at a regional level, but with considerably higher bias (−0.1782 to 0.0352 CLABSI/1000 CVC-days – Table 1). In addition, to yield 90% of valid iterations, the minimum participation required for adult ICUs was longer than at the national level (9 to 10 blocks), while the required participation for neonatal and pediatric ICUs was 9 and 12 to 13 blocks, respectively.

### Random scenarios with unequal ICU participation

Valid estimates of all annual ICU pooled CLABSI incidence rates were obtained in all scenarios (bias −0.0168 to 0.0054 CLABSI/1000 CVC-days –Table 1) at a national level. The average length of participation to obtain 90% of valid iterations was 3, 12, and 8 surveillance blocks for adult, pediatric, and neonatal ICUs, respectively.

At a regional level, valid estimates for all ICU CLABSI incidence rates were also obtained in all scenarios (bias −0.3745 to 0.0743 CLABSI/1000 CVC-days – Table 1). The average participation requirements for achieving 90% of valid iterations were longer for all types of ICUs (9 to 10 blocks for adult units, 13 blocks for PICUs, and 12 blocks for NICUs).

**Table 1:** Results of random data removal scenarios.

Scenario	Number of ICUs	Validity (%)	Average bias (per 1000 CVC-days)	Participation to reach 90% cut-off (surveillance blocks)
Equal participation				
*National*				
Adult ICUs	480	100	−0.0005 to 0.0004	3
PICUs	48	100	−0.0091 to 0.0105	13
NICUs	72	100	−0.0034 to 0.0119	13
*Regional*				
Adult ICUs	43	100	−0.0146 to 0.0213	9
PICUs	4	100	−0.1782 to 0.0186	12 (2007–2008) and 13 (2008–2009)
NICUs	6	100	−0.1387 to 0.0352	10
Unequal participation				
*National*				
Adult ICUs	480	100	−0.0003 to 0.0023	3
PICUs	48	100	−0.0168 to 0.0054	12
NICUs	72	100	−0.0040 to −0.00008	8
*Regional*				
Adult ICUs	43	100	−0.0162 to 0.0200	9 (2007–2008) and 10 (2008–2009)
PICUs	4	100	−0.3745 to 0.0742	13
NICUs	6	100	−0.2723 to 0.0145	12

ICU  =  intensive care unit; PICU  =  pediatric intensive care unit; NICU  =  neonatal intensive care unit; CVC-days  =  central venouscatheter-days.

### Non-random scenarios

**Figure 1:**
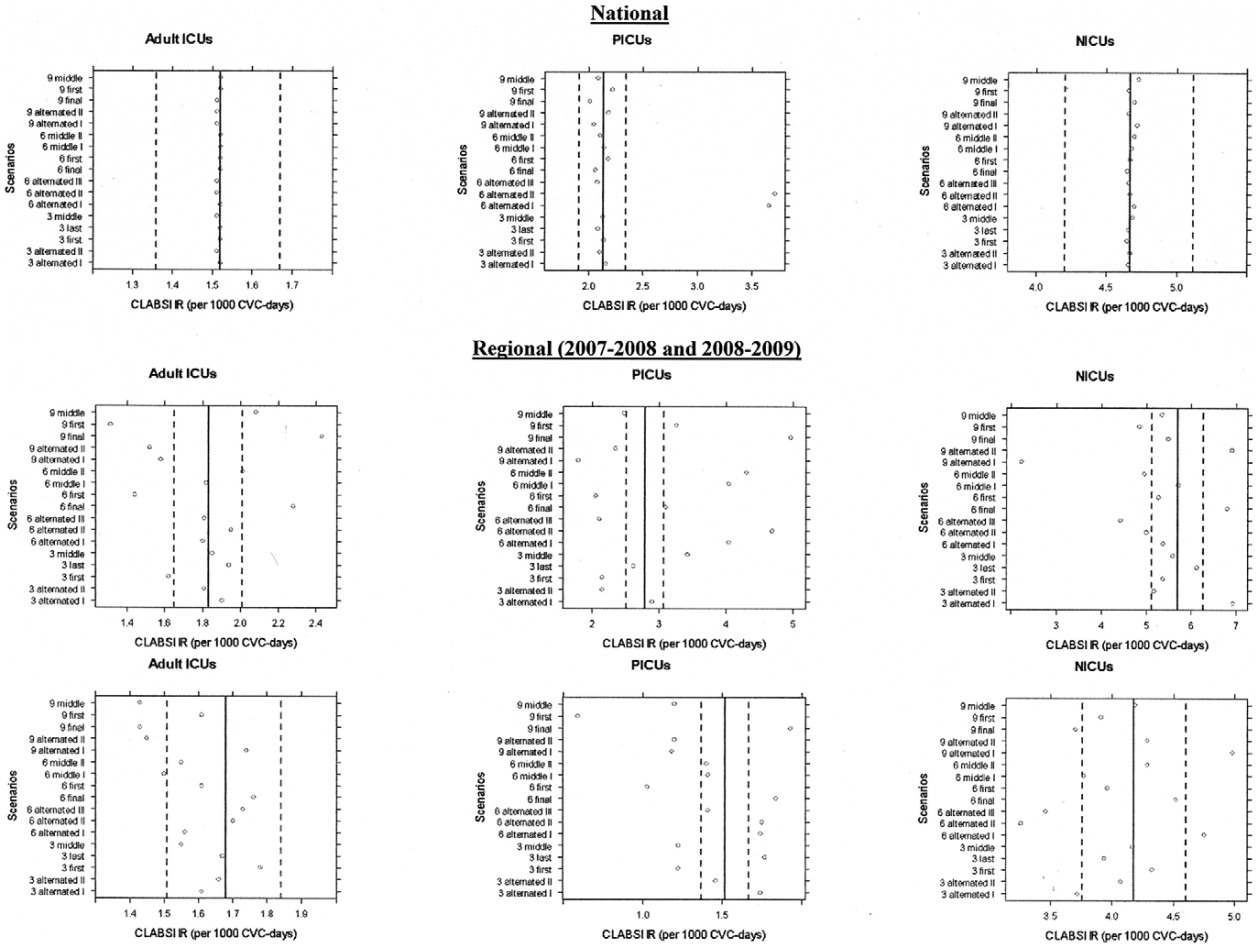
Graphic representation of the bias of the central line-associated bloodstream infection incidence rate estimates obtained for the non-random scenarios.

When using the national simulated database, the non-random scenarios that evaluated a total surveillance duration of 9 blocks per year generated estimates for adult ICU annual CLABSI pooled incidence rate that were valid for 100% of the sample of 100 simulated databases, while proportions of valid NICU and PICU estimates between 96 to 98%, and 68 to 78%, respectively. Overall, bias ranged from −0.0301 to 0.0337 CLABSI/1000 CVC-days (Figure 1). At a regional level, estimates of adult and neonatal ICU annual CLABSI pooled incidence rates were valid ≥80% of the time during the 2007–2009 period. PICUs presented the worst results, with only 40% (2007–2008) and 20% (2008–2009) of valid estimates. Overall bias was higher, ranging between −1.2291 and 0.6381 CLABSI/1000 CVC-days (Figure 1).

At a national level, scenarios in which surveillance lasted 6 blocks had 100% of estimates that were valid for adult ICU annual CLABSI pooled incidence rate, while NICUs presented proportions that varied from 84 to 92% (overall bias −0.035 to 0.015 CLABSI/1000 CVC-days). Less than 53% of PICUs estimates were valid, with much more prominent bias (−1.583 to 0.070 CLABSI/1000 CVC-days – Figure 1). When we calculated estimates using the regional database (bias from −1.9026 to 1.2666 CLABSI/1000 CVC-days – Figure 1), between 71% (2007–2008) and 86% (2008–2009) of estimates were valid for adult ICU annual CLABSI pooled incidence rates, while NICUs and PICUs presented validity proportions that were 43% (2007–2008) and 57% (2008–2009), and 43% (2007–2008) and 0% (2008–2009), respectively.

Finally, scenarios evaluating 3-block surveillance duration had the worst overall performance at both the national and regional levels. For national databases, while estimates for adult ICU annual CLABSI pooled incidence rate produced validity proportions of 97 to 99% and minimal bias (−0.0080 to 0.0068 CLABSI/1000 CVC-days), estimates for NICU and PICU were valid in 65 to 70% and 0 to 37% of cases, respectively, with bias between −0.0958 and 0.1118 CLABSI/1000 CVC-days (Figure 1). At a regional level, while the proportions of valid estimates for NICU annual CLABSI pooled incidence rate were 40% (2007–2008) and 60% (2008–2009), PICUs could not achieve valid estimates (0%) in any of the two years, and adult ICUs presented 40% of valid estimates during 2008–2009 and 0% during 2007–2008. Overall bias ranged between −2.1752 and 3.4614 CLABSI/1000 CVC-days (Figure 1).

## Discussion

Our study simulated the effect of different participation lengths on the validity of national and regional benchmarks for ICU annual CLABSI pooled incidence rates and demonstrated that surveillance programs should base their minimum participation requirements on the number of participating ICUs. If data are collected for random intervals during the year, it is possible to generate valid estimates of the true CLABSI incidence rates using less data. Nevertheless, this will only be achieved if a surveillance program has a high number of participating ICUs, as is the case for countrywide surveillance programs.

Our approach was to use all available data for the calculation of the annual CLABSI incidence rates, which is similar to what is currently done by regional and national surveillance programs worldwide. In our random scenarios, we assumed ICUs randomly chose when to collect data, something that may be achieved by asking units to determine a priori when data will be submitted to surveillance programs; e.g., before each surveillance block. In using this strategy, missing completely at random data were produced by design, which allowed the calculation of unbiased estimates of the annual ICU CLABSI pooled incidence rates.[14], [16], [17] However, as the estimates were calculated based on a lower number of observations, there was a loss in precision, which could be partially compensated for by either longer participation or a higher number of participants.

Based on our results, we recommend to maintain the requirement for continuous participation for small (<100 ICUs) CLABSI surveillance programs, i.e., regional programs, due to the limited number of participating ICUs. The elimination of continuous participation seems only suitable for national programs, with enough participating units to compensate for the reduction in surveillance duration. However, even national programs should be careful when doing so, as further stratification of CLABSI rates according to ICU types (e.g., adult cardiac or adult burn units), would cause a substantial decrease in sample size for incidence rate calculation, thereby threatening benchmark validity. This problem was exemplified by the lower precision and validity of PICU and NICU estimates compared to adult ICUs', which was driven not by different patient characteristics, but by the small number of participating units.

CLABSI incidence rates are assumed to vary randomly over the year, without a seasonal pattern.[18] Thus, as the monthly CLABSI incidence rate pattern may change over the years, it becomes problematic for surveillance programs to impose when participants should collect data. As shown in our non-random simulation scenarios for a regional surveillance program, options that worked relatively well in 2007–2008 did not have the same performance in 2008–2009 and vice-versa. Moreover, the validity of the results of non-random scenarios also seemed to be associated with sample size. Yet, despite presenting better results when used for the larger national surveillance program dataset, non-random strategy results were more unstable overall when compared to those produced by random scenarios. Therefore, we do not recommend the use of this strategy in either small or large surveillance programs.

Despite the fact that, according to our simulation models, the continuity of data collection might not be a prerequisite to obtain valid benchmarks at a countrywide level, we strongly advocate for continuous CLABSI surveillance throughout the year in all hospitals. At the hospital-level, annual CLABSI rates are very unstable because of the small number of CLABSI events and CVC-days. Therefore, missing 1 or 2 months of data can have a substantial impact on the annual rate, and cause important bias. In addition, one of the reasons for doing surveillance is to ensure the detection of outbreaks, something that is only achieved if rates are monitored in a continuous fashion.

This study's major limitation was the non-existence of a national CLABSI surveillance database where ICUs continuously participate throughout the year, as this forced us to simulate such a dataset. The use of the SPIN database to model the expected number of ICU CLABSI and CVC-days decreased the precision of our simulated results for PICUs and NICUs as shown by the random mean square error values for these units. This is due to the small number of available pediatric and neonatal units that exist in the Province of Quebec (5 and 7, respectively), and may partly explain the better performance in some of the scenarios of the real provincial database over our simulated one. However, as our objective was to simulate a range of plausible CLABSI incidence rates for adult, pediatric, and neonatal units, we were more interested in generating rates with low bias relative to the original SPIN rates, which was achieved, rather than with low variability. Also, for random scenarios, we assumed that ICUs randomly chose when to send data to the surveillance program, which may not be completely true. Due to feasibility issues common to all hospitals, it is possible that some ICUs will elect not to send data during periods in which the risk of acquiring CLABSI is higher, e.g., summer months, when nurse-patient ratio is reduced due to vacations and/or when a high number of new and inexperienced residents will be learning to insert CVCs in ICU patients, a phenomenon that was not accounted for in our model.[19], [20] Finally, due to the different sizes and ICU population characteristics of different regional and national surveillance programs, our results may not be widely generalizable.

Nevertheless, our study makes an important contribution to clarify the appropriateness of eliminating the continuous participation requirement from multicentre CLABSI surveillance programs. Although this strategy certainly decreases the financial burden of surveillance and therefore facilitates the recruitment and retention of participating hospitals, shortening the duration of surveillance performed per year may negatively impact the validity of the obtained results. To our knowledge, this is the first study that evaluates the effect of different surveillance programs' participation requirements on the validity of CLABSI incidence rate benchmarks. Problems arising from the generation of biased benchmarks are many, including the misleading of public health officers and infection control teams, who will not accurately identify priorities regarding CLABSI prevention and control.[21] Also, with the increased popularity of public reporting of healthcare-associated infection rates, biased benchmarks towards higher CLABSI incidence rates may worry stakeholders and the public about hospitals' performance from a region or a country.[22] Nevertheless, the major problem would arise from biased benchmarks towards lower rates, which could lead to a false belief in the success of the current infection control practices used to prevent CLABSI in ICUs.

In conclusion, our simulation models showed that the elimination of a continuous participation requirement may be a suitable alternative for large ICU CLABSI surveillance programs if data submitted are randomly collected. However, minimum participation length should be based on the number of participating ICUs, with smaller programs requiring longer participations. To decrease the risk of generating biased benchmarks, small surveillance programs for CLABSI in ICUs such as regional ones, should opt for continuous participation. Further research is needed to determine the optimal length of participation for different programs' size.

## Supporting Information

Appendix S1 Simulation involving a national CLABSI surveillance program.
(DOC)
