# Supplementary material for: Surveillance Length and Validity of Benchmarks for Central Line-Associated Bloodstream Infection Incidence Rates in Intensive Care Units
Source: PLoS One. 2012 May 7;7(5):e36582. doi: 10.1371/journal.pone.0036582 (PMC3346722; doi:10.1371/journal.pone.0036582)
Supplement: Appendix S1 — Simulation involving a national CLABSI surveillance program. (DOC) [file pone.0036582.s001.doc]

APPENDIX S1 - Simulation involving a national CLABSI surveillance program

Data sources

Two main data sources were used for modelling ICU population structure and individual ICU characteristics and annual CLABSI incidence rate:

- The Surveillance Provinciale des Infections Nosocomiales (SPIN) program database was used to provide data on the ICU population characteristics and on the expected number of CLABSI and CVC-days.[1-4] This is a provincial CLABSI surveillance program, which was launched in 2003 by the Institut National de Santé Publique du Québec and currently has 62 participating ICUs. Variables contained in this database include ICU type (adult, pediatric – PICU - and neonatal -NICU), number of beds, academic profile (“teaching” and “non-teaching units”), and annual number of CLABSI cases and CVC-days divided in 13 4-week surveillance periods. For this study, we used SPIN data for the period from April 2007 to March 2009, after participation in the program was determined to be mandatory for Quebec ICUs with ≥10 beds and the participating ICU population became more stable.
- The National Nosocomial Infections Surveillance (NNIS) System and the National Healthcare Safety Network (NHSN) were used to provide data on the characteristics of hospitals participating in a national CLABSI surveillance program. NNIS was the largest monitoring system for hospital-acquired infections in the U.S. until 2006, when it was replaced by the NHSN. In 2001, the NNIS group conducted a survey of a sample of 229 participating hospitals (81% of the total number of participants) in which the characteristics of hospitals, such as academic profile, were recorded.[5] The structure of the simulated ICU population regarding number of participating units and ICU type was derived from the 2006 NHSN report.[6]

Modelling simulated ICU characteristics

We simulated data collected by a countrywide ICU CLABSI surveillance program containing 600 ICUs (80% adult, 8% PICU and 12% NICUs) during a one-year period. Regarding academic profile, 60% of the simulated units were classified as “teaching ICUs”, defined as ICUs whose hospitals to which they belong are part of a teaching and research program of a medical school.[1] All simulation procedures were performed using R 2.11.1. We used the command “rbinom” to perform the random number generation for the binomial distribution of the variables “ICU type” and “academic profile” for each simulated ICUs, with the parameters size and probability.[7]

To determine the number of beds of the simulated units, we built a multivariate linear regression model using SPIN data for 2007-2009, which included the variables “academic profile” and “ICU type” The fitted equation used to model the ICU number of beds was:

ICU number of beds = 10.129 + 4.340*academic profile - 3.669*PICU + 10.103*NICU

Consequently, we used the command “rpois” to perform the random generation of numbers for the Poisson distribution of the variable “ICU number of beds”, with parameter lambda, i.e., a vector of non-negative means.[8]

Modelling number of CLABSI cases and CVC-days

Initially, using data from the SPIN database, we created a random effects Poisson model to model the number of CVC-days that would be observed in different ICUs in each of the 13 4-week surveillance blocks throughout a year. The model included the variables “academic profile”, “number of ICU beds”, and “ICU type”, and had random slopes for “surveillance blocks” and “ICU”. The command “rpois” was used to perform the random generation of values for the variable “CVC-days” for each of the 13 simulated surveillance blocks, and stored the results in a temporary vector.[8] The fitted equation used to predict the number of CVC-days for each simulated surveillance block was:

CVC-days = exp (3.10547 + 0.79459*academic profile - 1.31578*PICU - 1.21487*NICU + 0.07784*ICU beds)

Subsequently, we created a random effects Poisson regression model to predict the number of CLABSI cases that the simulated ICUs would present in each of the 13 surveillance blocks. The variables included in this second model were “academic profile”, “ICU type”, “number of ICU beds”, and the simulated variable “CVC-days”, this latter used as the offset. Again, the command “rpois” was used to randomly generate the values for the variable “CLABSI” for each of the 13 surveillance blocks. All results were stored in a temporary vector. The fitted equation used to model the CLABSI number per simulated surveillance block was:

CLABSI = exp (-6.69841 + 0.28540*academic profile + 0.33162*PICU + 1.11861*NICU - 0.00123*ICU beds) * CVC-days

REFERENCES

1. Fontela PS, Platt RW, Rocher I, et al. Surveillance Provinciale des Infections Nosocomiales (SPIN) Program: implementation of a mandatory surveillance program for central line-associated bloodstream infections. Am J Infect Control 2011;Jan 20 [Epub ahead of print].

2. Quach C, Frenette C, Gilca R, Rocher I, Moore D. Rapport de la surveillance provinciale des bactériémies nosocomiales sur cathéters centraux aux soins intensifs: avril 2006 - mars 2007. Montreal: Institut National de Santé Publique du Québec (INSPQ), 2007.

3. Quach C, Moore D, Rocher I, Frenette C. Rapport de la surveillance provinciale des bactériémies nosocomiales sur cathéters centraux aux soins intensifs: octobre 2003 - mars 2005. Montreal: Institut National de Santé Publique du Québec (INSPQ), 2005.

4. Quach C, Moore D, Rocher I, Frenette C, Gilca R. Rapport de la surveillance provinciale des bactériémies nosocomiales sur cathéters centraux aux soins intensifs: avril 2005 - mars 2006. Montreal: Institut National de Santé Publique du Québec (INSPQ), 2006.

5. Richards C, Emori TG, Edwards J, Fridkin S, Tolson J, Gaynes R. Characteristics of hospitals and infection control professionals participating in the National Nosocomial Infections Surveillance System 1999. Am J Infect Control 2001;29(6):400-3.

6. Edwards JR, Peterson KD, Andrus ML, et al. National healthcare safety network (NHSN) report, data summary for 2006, issued June 2007. Am J Infect Control 2007;35:290-301.

7. R Development Core Team. The binomial distribution. 2010 [cited 2011 24 March]; Available from: <http://127.0.0.1:23045/library/stats/html/Binomial.html>

8. R Development Core Team. The Poisson distribution. 2010 [cited 2011 24 March]; Available from: <http://127.0.0.1:23045/library/stats/html/Poisson.html>
